# The use of logic for machine learning models in sepsis

**DOI:** 10.1186/s40635-026-00921-5

**Published:** 2026-06-03

**Authors:** Danielle R. Lavage, Lauren N. Ward, Derek C. Angus, Jason N. Kennedy, Gregory F. Cooper, Christopher W. Seymour

**Affiliations:** 1 Department of Biomedical Informatics, University of Pittsburgh, Pittsburgh, PA USA; 2 Internal Medicine Critical Care Fellow, Department of Critical Care Medicine, University of Pittsburgh, 3550 Terrace St, Scaife Hall, Suite 600, Pittsburgh, PA 15213 USA

**Keywords:** Sepsis, Logic, Subtype

## To the editor:

Machine learning is increasingly used to identify subtypes, predict outcomes, and find optimal treatments in sepsis. Yet, the complexity of machine learning models may make them poorly suited for use by clinicians at the bedside, leading to challenges in adoption. New strategies are needed to create parsimonious models that are clinically interpretable but preserve predictive performance [1]. To address this gap, we tested how regression models using Boolean logic could identify clinical sepsis subtypes in three datasets compared to less parsimonious machine learning approaches.

## Methods

We previously performed a retrospective cohort study of electronic health records (EHRs) of adult patients (age > = 18 years) who met Sepsis-3 [2] criteria within 6 h at 12 UPMC hospitals in Southwestern PA [3]. A training set for the models contained 20,189 records from 2010 to 2012. The testing set contained 43,086 records from 2013–2014. For external validation, we studied a prospective cohort of 1,027 patients with clinically adjudicated Sepsis-3 at one academic institution from October 17, 2017 to June 6, 2019.

All patients were grouped into clinical sepsis subtypes using SENECA criteria, defined as *α*, *β*, *γ*, or *δ-type, that* were derived from 29 clinical variables in the EHR [3]. Euclidean distance was calculated to the published SENECA derivation subtype centroid, after which minimal distance identified a patient as *α*, *β*, *γ*, or *δ*-type.

To locate parsimonious predictor combinations, we employed logic regression models [1] with the primary outcome of assignment to the *δ*-type, the subtype most commonly associated with worse outcomes and differential treatment effects [2, 3]. To fit logic regression models with categorical variables, continuous clinical variables were dichotomized using Youden’s index (eTable S1), derived on the training set, with each demographic and biomarker variable fit independently against the binary δ-subtype outcome [4]. Next, logic regression and both logistic regression and elastic net were fit. Performance was assessed using area under the receiver operating characteristic curve (AUC) with bootstrapped 95% confidence intervals. Optimal logic trees were visualized to aid in interpretation. Brier scores measured calibration. All analyses used R software (version 4.3.1, R Core Team).

## Results

The validation cohort’s mean age was 59.3 (SD, 16.9 years), 46% were female, median Elixhauser Index was 3.2 (SD, 2.1), and sequential organ failure assessment score on arrival was 3 [IQR: 2, 5] (Table 1). Age, sex, and illness severity were similar in training and test data. In-hospital mortality was 10%, 6%, and 9.5% (training, test, validation, respectively). The delta type was least common in all three datasets (13%, 14%, and 14%, training, test, validation, respectively).

**Table 1:** Patient characteristics

Variable	Training data	Test data	External validation data
No. of patients (%)	20,189	43,086	1,027
Age, mean (SD), y	64 (17)	67.5 (16.8)	59.3 (16.9)
Male, no. (%)	10,022 (50)	21,993 (51)	554 (54)
Race: no. (%)^a^			
White	15,640 (78)	36,820 (86)	950 (92.5)
Black	2,428 (12)	5,008 (12)	69 (6.7)
Other	2,121 (11)	1,258 (3)	8 (0.8)
Elixhauser, mean (SD)^b^	1.8 (1.2)	1.2 (1.2)	3.2 (2.1)
**Sepsis episode**			
Alpha-type, no. (%)	6625 (33)	12,485 (29)	349 (34)
Beta-type, no. (%)	5512 (27)	12,508 (29)	310 (30)
Gamma-type, no. (%)	5385 (27)	12,121 (28)	221 (22)
Delta-type, no. (%)	2667 (13)	5972 (14)	147 (14)
Surgery during hospitalization, no. (%)	2,727 (14)	5,122 (12)	NA
SOFA score, median (IQR)^c^	3 (2–5)	3 (2–4)	3 (2–5)
SIRS criteria, median (IQR)^d^	2 (1–3)	1 (1–2)	NA
Troponin, median (IQR), ng/mL	0.1 (0.04–0.11)	0.04 (0.04–0.1)	0.1 (0.1–0.25)
Serum lactate, median (IQR), mmol/L	1.5 (1–2.4)	1.5 (1–2.3)	1.4 (0.95–2.4)
Platelet count, median (IQR), × 10^3/µL	188 (130–256)	192 (134–260)	213 (150–293)
INR, median (IQR)	1.3 (1.1–1.6)	1.2 (1.1–1.7)	1.2 (1.1–1.5)
Total bilirubin, median (IQR), mg/dL	0.8 (0.5–1.3)	0.68 (0.4–1.1)	0.7 (0.5–1.4)
Mechanical ventilation, no. (%)	5,773 (29)	2,157 (5)	238 (23.2)
Total hospital vasopressor days, mean (SD)	4.3 (4.9)	0.5 (2.0)	2.7 (1.8)
In-hospital mortality, no. (%)	2,082 (10)	2,666 (6)	98 (9.5)

ICU, intensive care unit; SD, standard deviation; SOFA, Sequential Organ Failure Assessment; IQR, interquartile range; SIRS, systemic inflammatory response syndrome; INR, international normalized ratio

SI conversion factors: to convert serum creatinine to micromoles per liter, multiply by 88.4; platelet count to X10^9^/L, multiply by 1.0; and total bilirubin to micromoles per liter, multiply by 17.104

^a^Derived from UPMC registration system data using fixed categories similar to the Centers for Medicare & Medicaid Services electronic health record meaningful use data set. “Other” included American Indian or Alaska Native

^b^An overall measure of comorbidity burden derived from diagnostic codes at hospital discharge [5]

^c^A measure of acute organ dysfunction, assessed across 6 organs, with scores ranging from 0 to 24, maximum score reached within 6 h of sepsis episode

^d^A measure of systemic inflammation, ranging from 0 to 4, maximum score reached within 6 h of sepsis episode

**Fig. 1:**
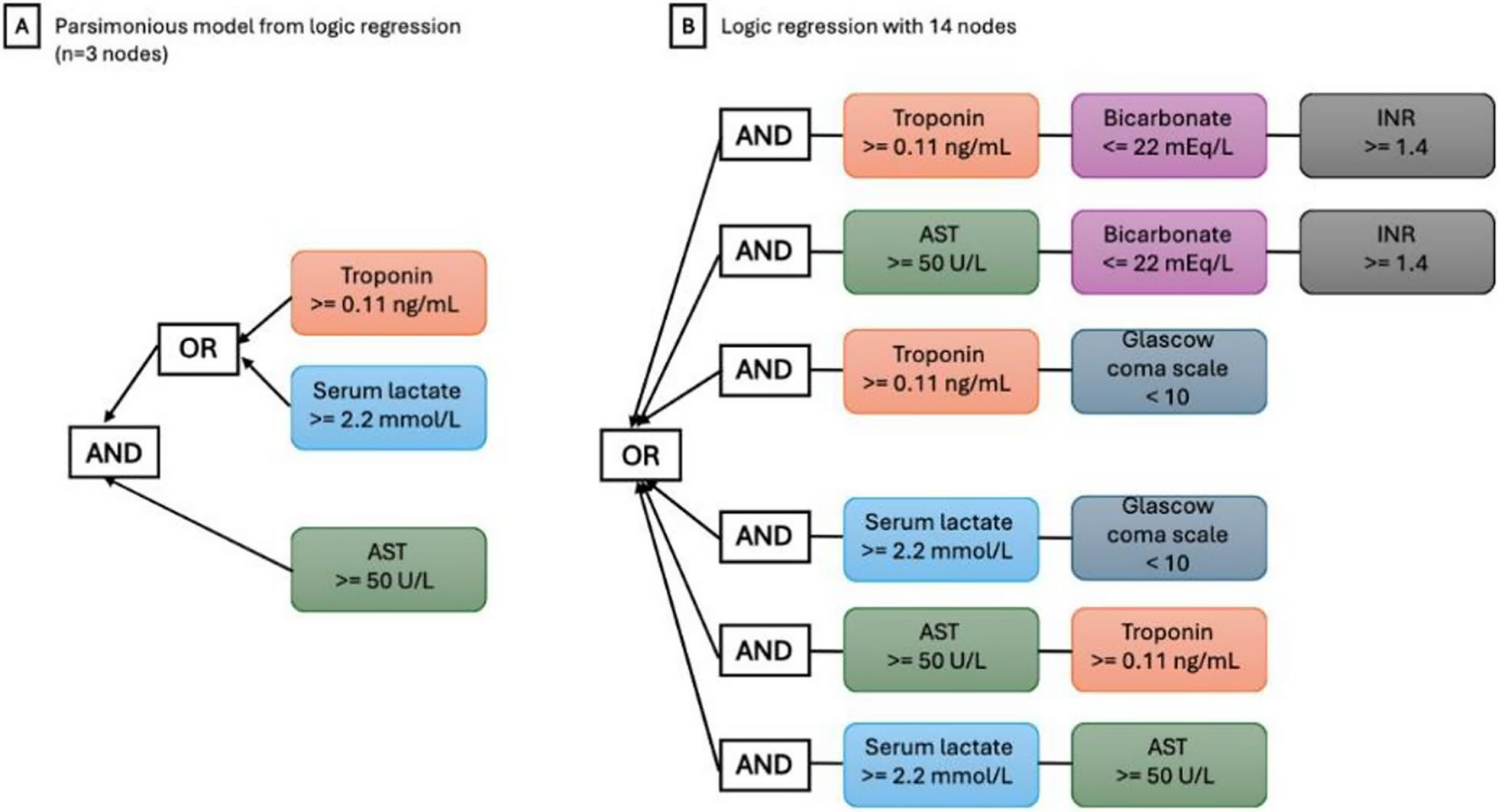
Logic regression examples with multiple nodes (panel **A**, 3 nodes; panel **B**, 14 nodes). Logic regression examples with multiple nodes. Panel **A** shows a very parsimonious logic regression model with only 3 nodes. Panel **B** shows a more expansive logic regression model with 14 nodes, of which 6 nodes are unique. The models shown in both panels were derived on all 29 available variables

The best logic regression model had 6 variables and 14 nodes (Fig. 1, Panel B). Delta-type prediction was accurate in both test AUC = 0.83 (95% CI: 0.83, 0.84), sensitivity/specificity = 0.9/0.77 and validation data 0.79 (0.76, 0.83), 0.81/0.78. A parsimonious model (Fig. 1, Panel A) contained only 3 variables (AST > = 50 U/L AND (lactate > = 2.2 mmol/L or troponin > = 0.11 ng/mL)) and had AUC = 0.77 (95% CI: 0.77, 0.78), sensitivity/specificity = 0.94/0.61. Fully parameterized elastic net and multivariable regression models both had AUC = 0.96 (0.96, 0.96) in the test data. Calibration of the model on test data was adequate (prevalence of 13.9%, scaled brier score of 0.33).

## Discussion

In multiple datasets of patients with sepsis, more complex machine learning models to identify subtypes can be simplified using logic regression while attaining strong predictive performance. Both multivariate logistic regression and elastic net had higher AUCs than logic regression. However, the multivariate models used all variables, and thus, understanding such models may be more difficult than the logic regression models that contain a small subset of the domain variables. Such a tradeoff of understanding the clusters and predicting them may increase as the modeling method becomes increasingly complex. Using all the variables the original sepsis subtype was developed on can also explain the high AUCs presented in the logistic and elastic net models. Variables located by the logic regression models are coherent across our model fitting. For instance, AST, lactate and troponin appear in every model after they first appear. This suggests reliability of variable detection across the models built. In comparison with the fully parameterized model, the most parsimonious model does have a lower AUC, but still provides an interpretable model for easier identification of δ-subtype patients, especially when considering resource-limited clinical settings. Furthermore, the 3 variables included in this model highlight the greater levels of liver dysfunction, serum lactate, and troponin observed in δ-subtype patients.

A limitation of the study is that though the proportion of δ-type subtype is similar across datasets, mortality, vasopressor use, and mechanical ventilation rates vary, which may affect the models’ performance. These differences may be related to the different time periods the training, testing, and validation datasets were collected from and potentially reflect changes in clinical practice patterns and coding over a 9-year span, thus introducing possible unmeasured confounding variables. However, the use of datasets across separate time periods also allows for temporal validation to improve the robustness of the model.

Another limitation of the study is that the explainability of the models was not assessed quantitatively, although logic regression is inherently interpretable. Whether such models are adaptable to clinical bedside care is beyond the scope of this study. Nonetheless, the predictive performance of parsimonious subtype models using logic regression suggests that investigating bedside implementation is warranted.

## Supplementary Information

Additional file 1.

## Data Availability

The datasets used and/or analyzed during the current study are available from the corresponding author on reasonable request.
