# Supplementary material for: The use of logic for machine learning models in sepsis
Source: Intensive Care Med Exp. 2026 Jun 3;14:66. doi: 10.1186/s40635-026-00921-5 (PMC13234080; doi:10.1186/s40635-026-00921-5)
Supplement: Supplementary file 1 — Additional file 1. [file 40635_2026_921_MOESM1_ESM.docx]

**eTable 1 (Supplemental)** Youden’s Index Cut Points

| **Variable** | **Direction of Positive Indication^a^** | **Youden’s Cut Point^b^** |
| --- | --- | --- |
| Age, years | <= | 76 |
| Albumin, g/dL | <= | 2.8 |
| Alanine transaminase, U/L | >= | 48 |
| Aspartate transaminase, U/L | >= | 50 |
| Premature neutrophil count (bands), % | >= | 8.5 |
| Bicarbonate, mEq/L | <= | 22.8 |
| Total Bilirubin, mg/dL | >= | 1.4 |
| Blood urea nitrogen (BUN), mg/dL | >= | 25 |
| Chloride, mEq/L | >= | 106 |
| Creatinine, mg/dL | >= | 1.52 |
| C-Reactive Protein, mg/L | >= | 9.1 |
| Erythrocyte sedimentation rate, mm/h | <= | 43 |
| Glasgow Coma Scale score | <= | 10 |
| Glucose, mg/dL | >= | 175 |
| Hemoglobin, g/dL | <= | 12.1 |
| Heart Rate, beats/min | >= | 105 |
| International normalized ratio | >= | 1.4 |
| Serum Lactate, mmol/L | >= | 2.2 |
| Partial pressure of oxygen, mm Hg | >= | 102 |
| Platelet Count, x10^3/µL | <= | 136 |
| Respiration Rate, breaths/min | >= | 23 |
| Oxygen Saturation, % | >= | 94.9 |
| Sodium, mEq/L | >= | 140 |
| Systolic BP, mm Hg | <= | 97 |
| Temperature, °C | <= | 36.22 |
| Troponin, ng/mL | >= | 0.11 |
| White blood cell count, x10^9/L | >= | 12.8 |

a Values indicated by the direction in table suggest positive relationships with subtype delta. For instance, patients with AST >= 50 were more likely to be subtype delta than those note. These records were then mapped to positive binary values.

b Youden’s index was derived for each variable independently using logistic regression models to find the optimal value for each biomarker. Models were fit on the training set only and used the delta subtype as the outcome.
